# Developing Functional Relationships between Soil Waterlogging and Corn Shoot and Root Growth and Development

**DOI:** 10.3390/plants10102095

**Published:** 2021-10-03

**Authors:** Charles Hunt Walne, K. Raja Reddy

**Affiliations:** Department of Plant and Soil Sciences, 117 Dorman Hall, Box 9555, Mississippi State University, Mississippi State, MS 39762, USA; chw148@msstate.edu

**Keywords:** corn, flooding, functional relationships, maize, modeling, root growth, shoot growth, waterlogging, modeling

## Abstract

Short- and long-term waterlogging conditions impact crop growth and development, preventing crops from reaching their true genetic potential. Two experiments were conducted using a pot-culture facility to better understand soil waterlogging impacts on corn growth and development. Two corn hybrids were grown in 2017 and 2018 under ambient sunlight and temperature conditions. Waterlogging durations of 0, 2, 4, 6, 8, 10, 12, and 14 days were imposed at the V2 growth stage. Morphological (growth and development) and pigment estimation data were collected 15 days after treatments were imposed, 23 days after sowing. As waterlogging was imposed, soil oxygen rapidly decreased until reaching zero in about 8–10 days; upon the termination of the treatments, the oxygen levels recovered to the level of the 0 days treatment within 2 days. Whole-plant dry weight declined as the waterlogging duration increased, and after 2 days of waterlogging, a 44% and 27% decline was observed in experiments 1 and 2, respectively. Leaf area and root volume showed an exponential decay similar to the leaf and root dry weight. Leaf number and plant height were the least sensitive measured parameters and decreased linearly in both experiments. Root forks were the most sensitive parameter after 14 days of waterlogging in both experiments, declining by 83% and 80% in experiments 1 and 2, respectively. The data from this study improve our understanding of how corn plants react to increasing durations of waterlogging. In addition, the functional relationships generated from this study could enhance current corn simulation models for field applications.

## 1. Introduction

Waterlogging is a severe abiotic stress occurring when the soil profile surrounding a plant’s root system becomes oversaturated with excess water. Waterlogging is part of the broader stress of flooding, which encompasses both situations where the soil profile is oversaturated and when visual ponding occurs above the soil surface [1]. Waterlogging can occur anytime soil moisture levels rise above the field capacity. Water inputs exceed a soil’s ability to move water off the soil surface and drain internally. Excessive moisture levels result in the aerated pore space filling with water. A water-filled pore space leads to soil oxygen levels rapidly depleting as the transfer of oxygen and other gasses is blocked between the soil and the atmosphere. Oxygen diffuses 10,000 times more slowly in water than in air [2,3].

Excess moisture can come from extreme rainfall events, over-irrigation, and rainfall occurring soon after irrigation, among other factors [4]. Poor soil structure caused by natural or human influences may also restrict the internal drainage within the soil and further worsen waterlogging [5]. As the soil remains waterlogged, the soil redox potential declines [6], and the accumulation of reduced substances can reach phytotoxic levels [7]. Additionally, soil pH has been reported to increase due to protons’ consumption by reducing Fe and Mn oxides [6]. However, Drew et al. [8] suggest that the shortage of the oxygen available to plant roots is still the main factor restricting plant productivity.

Globally, waterlogging restricts an estimated 10–12% of all agricultural lands annually [9], and flooding affects over 17 million km^2^ of land surface [10]. Waterlogging ranked second to drought in the United States in terms of abiotic stress’ contribution to crop production losses from 2000 to 2017 [1,11]. Early research suggests that 15.7% of US agricultural soils are impaired by wet conditions, and 16.4% of crop insurance payments from 1939 to 1978 resulted from excessive water [12]. Despite waterlogging appearing as a significant limiting factor to agriculture throughout history, the harmful impacts on production have worsened following increases in the frequency of extreme precipitation events over the past 50 years [1]. These extreme precipitation events include a general upward trend of total precipitation correlated with an increased frequency of heavy and extreme precipitation events [13]. Global climate models consistently forecast these trends into the future [14]. Specifically in the Lower Mississippi River Alluvial Valley (LMRAV), increasing trends in annual precipitation and mean wet days are occurring [15]. For example, an increase in spring precipitation was reported in the northern LMRAV; conversely, precipitation in the southern LMRAV is increasing more in the fall, often related to hurricane formation [15]. As target-planting dates for corn are pushed earlier into the spring, corn will be increasingly vulnerable to extreme moisture levels during early vegetative growth stages.

Previous research outlines waterlogging as a significant yield-limiting factor for corn production. Singh et al. [16] indicated that yields in low-lying topographic areas were significantly lower in years when waterlogging occurred. Waterlogging for 10 days has been reported to significantly reduce yields, dry matter accumulation, and canopy height [17]. Field studies have suggested a 4.69% decline in yield for each day of waterlogging up to seven days, compared to the non-flooded control [3]. Research has also established that corn is more vulnerable at early growth stages; Ren et al. [18] report a more significant yield loss occurring from six days of waterlogging occurring at V3 than V6 and VT.

Although the research indicates the damaging effects of waterlogging on corn yield, no systematic evaluation of the impact of various waterlogging durations on corn morphology has been conducted to our knowledge. A systematic study will help unravel the relationships between the duration of waterlogging stress, soil oxygen levels, and corn morphological growth and development. In addition, field studies examining the impact of waterlogging on corn production can be influenced by confounding factors such as nitrogen availability post waterlogging. The same rainfall events that cause waterlogging can disrupt nitrogen availability through runoff and leaching. The low oxygen environment arising under waterlogging conditions also creates a favorable environment for further nitrogen loss through denitrification. For example, Kaur et al. [3] concluded that additional rescue N applications at V10 helped reduce some yield reduction compared to non-treated controls when plants were waterlogged for seven days at the V6 growth stage. Therefore, identifying and isolating the impact of waterlogging on corn morphological dynamics would be best conducted under conditions in which plant nutrients are freely available and non-limiting for plant uptake.

The objectives of this study were to: (1) quantify the effects of increasing durations of waterlogging from 0 to 14 days occurring at an early vegetative growth stage on shoot and root morphology, (2) determine relationship trends between waterlogging duration, soil O_2_, and growth and development parameters, and (3) compare the magnitude of the impact of waterlogging stress among all measured growth and development parameters.

## 2. Materials and Methods

### 2.1. Experimental Facilities

Two experiments (Experiment 1, Exp. 1; Experiment 2, Exp. 2) were conducted outdoors in pots during the 2017 and 2018 growing seasons at the Environmental Plant Physiology Laboratory at the Mississippi Agricultural and Forestry Experiment Station, Mississippi State University, MS, USA (33°28′ N, 88°47′ W). The pot-culture facility includes 30.5 × 15.2 cm polyvinylchloride (PVC) pots constructed with a small drain hole at the bottom. The pots were arranged in a twin-row configuration with extra border pots on both ends. A drip irrigation system combined with a customized nutrient solution provided a precise control over soil moisture and nutrition. Rain shelters were constructed as mini hoop-houses and placed over the pots during the experimental period to ensure outside precipitation did not affect the experiment. These rain shelters were constructed with clear plastic not to inhibit photosynthetically active radiation. In addition, they were well ventilated to prevent extreme temperature elevation. The environmental conditions, including temperature, CO_2_ level, and solar radiation, under which each experiment was conducted are provided in Table 1.

### 2.2. Experimental Set-Up

Six polyvinyl (PVC) pots (15.24 cm diameter, 30.48 cm height, and 5.5 L volume) per treatment were initially filled with one inch of clean pea gravel, 500 g gravel to aid drainage. The pots were then filled with growth media consisting of a 3:1 ratio of fine sand (particle size less than 0.3 mm) and ground topsoil. The soil medium is sandy loam with 87% sand, 2% clay, and 11% silt. Four seeds were initially sown in each pot. Upon emergence, the plants were thinned to one plant per pot. Each pot was irrigated with a full-strength Hoagland’s complete nutrient solution [19] thrice per day for 90 s throughout the experiment to ensure that the pots were maintained at field capacity. This solution ensures that all necessary nutrients are readily available for plant uptake. Similar pot culture facilities and methods have been used to study soybean and moisture stress [20], rice and moisture stress [21], rice and salinity stress [22], cowpea and drought stress [23], and sorghum and nitrogen stress [24].

### 2.3. Plant Materials

Two corn hybrids were tested during this study. Pioneer P2089VYHR (Corteva, Inc., Wilmington, DE, USA) was grown in the first experiment, and Agrigold A6659 (Agrigold Inc., St. Francisville, IL, USA) was grown in the second.

### 2.4. Waterlogging Treatments

Soil moisture was maintained at optimal field capacity until the plants reached the second leaf (V2) growth stage. Waterlogging treatments were imposed by plugging the drain hole at the bottom of each pot with a small wooden peg. The treated pots were filled to the soil surface with the Hoagland’s nutrient solution. Water levels were continuously monitored twice each day to ensure that the treatments were continuously maintained for the appropriate assigned duration. Apogee SO-110 soil oxygen sensors (Apogee Instruments, Inc., Logan, UT, USA) were placed into three randomly selected pots per treatment to continuously monitor the soil oxygen level and temperature throughout the experimental period. Once the duration of treatment had passed, the wooden drain plug in the respective experimental unit was removed, and the pot was fully drained. Soil moisture was then maintained at field capacity for the duration of the experiment. The treatments included a 0 days treatment, which we considered the control treatment, displaying the plant’s potential under non-waterlogged conditions, and 2, 4, 6, 8, 10, 12, and 14 days of waterlogging.

### 2.5. Measurements

The measurements detailed below were collected at the final harvest, 15 days after the treatments were initially imposed (DAT) and 23 days after the seeds were sown (DAS).

#### 2.5.1. Pigment Estimation

The leaf chlorophyll content (CHL, μg/cm^2^), flavonoids (FLAV, unitless), anthocyanins (ANTH, unitless), and nitrogen balance index (NBI, unitless) were measured on the uppermost leaf with a developed collar using a handheld Dualex^®^ Scientific instrument (Force A DX16641, Paris, France).

#### 2.5.2. Shoot Morphology

Plant height (PH, cm plant^−1^) was measured by hand with a standard metric ruler as the distance from the soil surface to the highest leaf collar. Leaf number (LN, no. plant^−1^) was counted as the total number of fully developed leaves with a collar. The plants were then cut at the soil surface; all leaf material was separated from the stem and measured for leaf area (LA, cm^2^ plant^−1^) using an LI-3100 leaf area meter (LiCor Inc., Lincoln, NE, USA).

#### 2.5.3. Root Morphology

After the above-ground plant parts were removed, root systems and soil media were gently removed from the PVC pots and washed with a gentle stream of water over a wire mesh sieve to remove soil media until the roots were clean. Individual root systems were floated in a 400 × 300 cm acrylic tray filled with 5 mm water. Once placed on the trays, the roots were carefully untangled using plastic forceps to minimize roots’ overlap to ensure quality imagery. An Epson Expression 11000XL scanner captured root morphology images at a 800 dpi resolution (Epson America, Inc., Long Beach, CA, USA). These images were analyzed by WinRHIZO Pro 2009C software (Regent Instruments, Inc., Québec, QC, Canada). The digitized output from the analysis quantified the multiple root growth and development parameters for each plant: root tips (RT, no plant^−1^), root forks (RF, no plant^−1^), total root length (TRL, cm plant^−1^), root surface area (RSA, cm^2^ plant^−1^), and root volume (RV, cm^3^ plant ^−1^)

#### 2.5.4. Biomass Allocation

After the plant components were analyzed to the extent aforementioned, separate plant parts were individually bagged. The samples were oven-dried on-site at 80 °C for three days to ensure a constant weight was reached. These samples were weighed for leaf dry weight (LDW, g plant^−1^), stem dry weight (StDW, g plant^−1^), and root dry weight (RDW, g plant^−1^). The total dry weight (TDW, g plant^−1^) was calculated as the sum of all three components per plant.

### 2.6. Waterlogging Stress Response Index

A waterlogging stress response index (WSRI) was calculated for each measured parameter to normalize the impact of flood stress on a comparable scale. This index was derived from the environmental productivity index (EPI) concept initially introduced [25] to model the impact of multiple environmental stresses on cactus productivity. Reddy et al. [26] also used the EPI concept to model the effect of multiple environmental stresses on cotton (*Gossypium hirsutum*) photosynthesis.

This study modified the EPI concept to model the waterlogging impact on multiple plant growth and development traits. The WSRI concept involves normalizing each parameter’s mean-observed values by dividing the mean values under stress by the value of the respective parameter under the 0 days treatment or control conditions. Thus, the indices’ values range from 1, where waterlogging stress is not limiting the parameter, to 0, where waterlogging is fully limiting. We can use this method to normalize data to compare waterlogging’s impact among parameters measured with different units. The values portray a fractional value of maximal potential performance for each parameter of each hybrid. The index values were derived using Equation (1).

WSRI*_pt_* = p*_t_*/p_c_ (1)

where WSRI*_pt_* is the waterlogging stress response index of parameter *p* at treatment *t*, p*_c_* is the value of parameter *p* at 0 days of waterlogging (control), and p*_t_* is the parameter *p* at treatment *t*.

### 2.7. Statistical Analysis

In both experiments, a completely randomized design (CRD) was utilized. The treatments were assigned to experimental units (pots) completely randomly in both experiments. In addition, this study included the use of two different hybrids, with each utilized in a separate experiment. Thus, the analysis was conducted as two independent experiments, and no direct statistical comparisons were made between the two hybrids. A similar experimental design and analysis were performed by Reddy et al. [27] to model the effects of UV-B radiation on two separate corn genotypes.

The analysis of variance testing was completed using the PROC GLM procedure in SAS 9.2 to determine the significance of treatment effects. Treatment means were separated using Fisher’s LSD at an alpha level of 0.05 (SAS Institute, Cary, NC, USA). Graphing and graphical functions were created using SigmaPlot 13 (Systat Software, Inc., San Jose, CA, USA).

## 3. Results and Discussion

**Figure 1:**
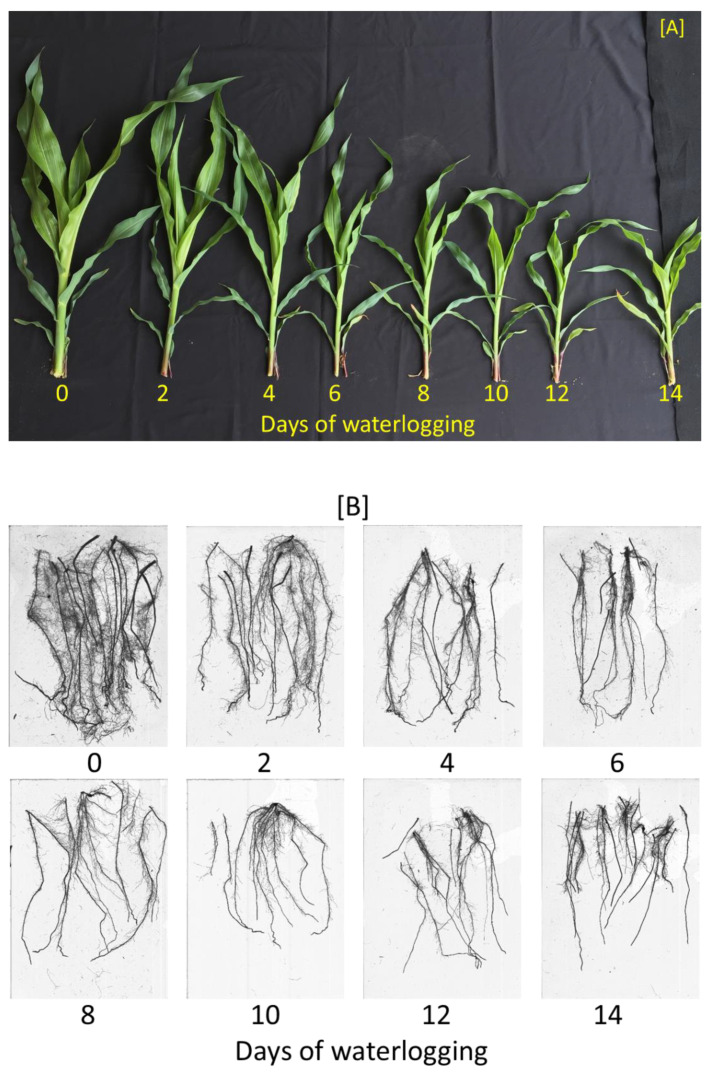
Pictorial representation of waterlogging effects on corn (**A**) shoot and (**B**) root growth during Experiment 1. The pictures were taken at the final harvest, 15 days after treatment, 23 days after planting.

In both experiments, soil waterlogging treatments significantly affected all plant growth, development, and physiology parameters except for the root: shoot ratio and flavonoids index (Table 2). Visually, as the waterlogging duration increased, the plants were stunted with smaller and fewer leaves (Figure 1A). In addition, below the soil surface, increasing the waterlogging durations caused the root systems to appear thinner and shallower (Figure 1B).

### 3.1. Environmental Conditions and Soil Oxygen

**Figure 2:**
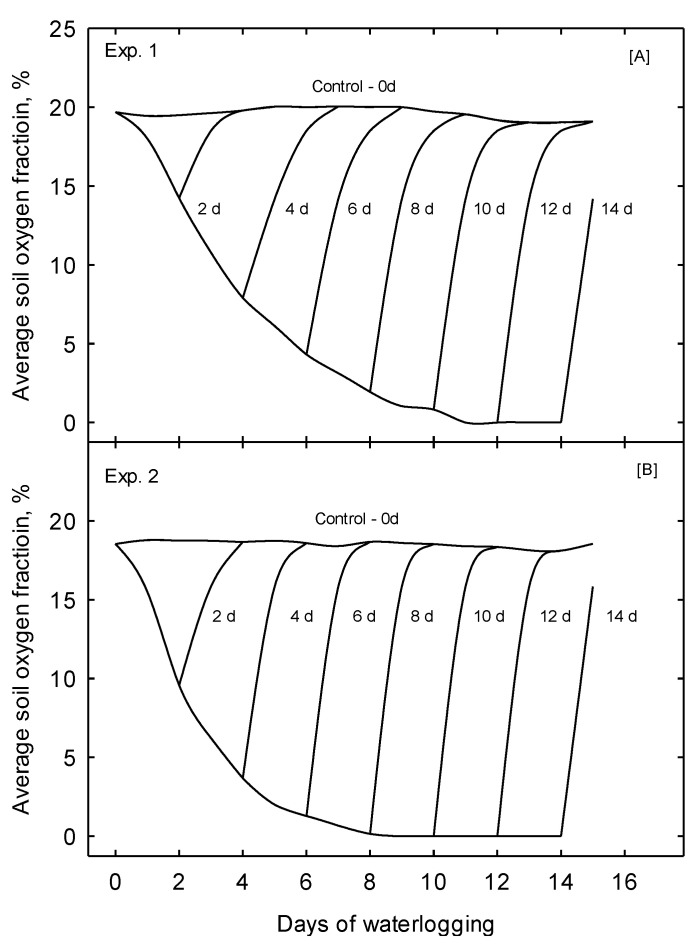
Changes in the soil oxygen fraction ((**A**)—Expt. 1 and (**B**)—Expt. 2) concerning the time after the treatments were imposed. Three pots were randomly selected from each treatment to monitor the soil oxygen fraction continuously.

During the experimental period of both experiments, the treatments significantly decreased the soil oxygen fraction (Figure 2). Pots subjected to the 0 days treatment, which we considered the control, maintained a steady soil O_2_ fraction throughout the experimental period. The average soil O_2_ fraction for the 0 days treatment was 19.6 for Experiment 1 (Exp. 1) and 18.54 for Experiment 2 (Exp. 2). As treatments were terminated at the end of each treatment period, the pots rapidly drained, and the soil O_2_ fraction quickly recovered. Oxygen approached the equivalent level of the 0 days treatment within 2 days of respective treatment termination. Overall, the soil O_2_ fraction was lower for the 0 days treatment for Exp. 2 and declined faster as the treatment duration was prolonged. The discrepancy of the soil O_2_ fraction and the decline rate between the two experiments may be due to the observed differences in air temperature, soil temperature, and solar radiation during the two experimental periods (Table 1). Specifically, air temperature and soil temperature were warmer, and daily solar radiation was higher during Exp. 2.

Further, the warmer environmental conditions may explain the higher growth potential observed under the 0 days treatment in Exp. 2. However, due to the experimental design of this study, the effects of different hybrids cannot be statistically separated from the impact of environmental conditions and their interaction. Therefore, each experiment was independently analyzed, and no direct comparisons between the hybrids were made.

### 3.2. Root Morphology

Root systems are directly impacted when waterlogging occurs, as they are the first to suffer from oxygen deprivation [28,29]. As soil oxygen levels begin to deplete, plants can signal the decline and slow their metabolism as a rescue mechanism to reduce the plant’s demand for oxygen [2]. When oxygen levels further decline and become more hypoxic, the respiration in the root system begins to switch from aerobic to anaerobic fermentation in an attempt to maintain an energy supply as oxygen supplies diminish [7]. Unfortunately, anaerobic fermentation produces less energy than aerobic respiration, and toxic byproducts accumulate within plant cells. These toxins become harmful, as they contribute to the injury and death of root cells and root decay [7,8].

#### 3.2.1. Root Growth

Total root length (TRL), root surface area (RSA), and root volume (RV) represent the vastness of soil the root system can reach and the total surface in which water and nutrient uptake can occur [30]. A plant’s productivity is directly related to its root system’s ability to explore the soil and forage for moisture and nutrients (root length) and its morphological capacity to uptake the available water and nutrients (root surface area).

**Figure 3:**
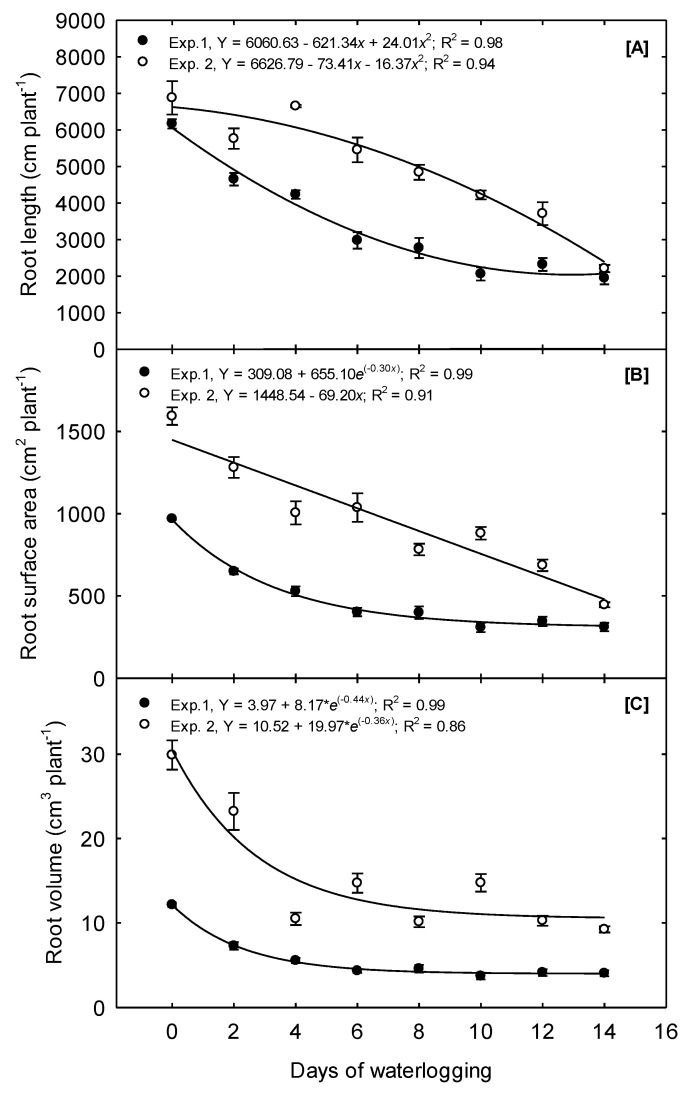
Waterlogging effects on corn root growth measured 15 days after waterlogging treatment initiation. (**A**) Mean root length showing a quadratic and convex quadratic decay in Exp. 1 and Exp 2, respectively. (**B**) Mean root surface area shows exponential and linear decay in Exp. 1 and Exp. 2, respectively. (**C**) Mean root volume shows the exponential decline in both experiments. The values are the mean of six replications for each treatment.

In Exp. 1, just two days of waterlogging resulted in a 24.5% reduction in the total root length (Figure 3A). Overall, the total root length quadratically declined as waterlogging treatments increased from 0 to 14 days. The steepest decline occurred as waterlogging increased from 0 to 8 days, and no differences in TRL occurred between the 10, 12, and 14 days treatments (Table 2). The response of TRL to waterlogging treatments differed in Exp. 2; a convex quadratic function best described the relationship to waterlogging duration, with the most significant decline in TRL occurring as the treatments increased from 6 to 14 days.

The RSA per plant decreased quadratically in Exp. 1 as treatments were prolonged from 0 to 6 days (Figure 3B). The final root surface area was reduced by 33%, 58.5%, and 67.9% because of 2, 6, and 14 days of waterlogging, respectively, compared to the 0 days treatment. No differences occurred due to the 6 to 14 days treatment; thus, linear decay functions best described the overall relationship between RSA and waterlogging duration (Table 2). In Exp. 2, the root surface area declined linearly as waterlogging time was increased from 0 to 14 days at a rate of 4.4% day^−1^. The highest RSA, of 1592, was observed under the 0 days treatment, and the lowest, of 446, was observed under the 14 days treatment, a total decline of 72%.

The RV in Exp. 1 followed trends similar to those observed for RSA. The RV declined from its highest value of 12.15 under the 0 days treatment to 4.32 under the 6 days treatment, a decrease of 64% (Figure 3C). No differences were observed in the mean RV between the 6 and 14 days treatments (Table 2); therefore, the exponential decay functions best described the overall relationship. In Exp. 2, RV declined linearly as the treatment increased from 0 to 4 days at a rate of 16.25% day^−1^. The mean RV did not significantly differ among the 4 to 14 days treatments. An exponential decay function also best described the overall relationship between RV and waterlogging in Exp. 2.

Our results agree with Ren et al. [18], who reported a decrease in the corn root absorption area after six days of waterlogging and expand upon their results to suggest that the severity of the decline in the root absorption area depends on the duration of waterlogging.

#### 3.2.2. Root Development

Root tips are critical components for nutrient and water uptake; the uptake occurs more predominantly near the tips due to a higher expression of nutrient transporters and water channels than in other parts of the root structure [31]. Therefore, root tips are also particularly susceptible to hypoxic conditions. If anaerobic conditions remain too long, cell death in the root tips can occur due to cytoplasmic acidosis caused by the lactic acid synthesis of anaerobic respiration. This can result in permanent damage to the plant. However, if hypoxic conditions gradually precede anoxia, corn root tips have been shown to survive continuous anoxia for up to 4 days [8].

**Figure 4:**
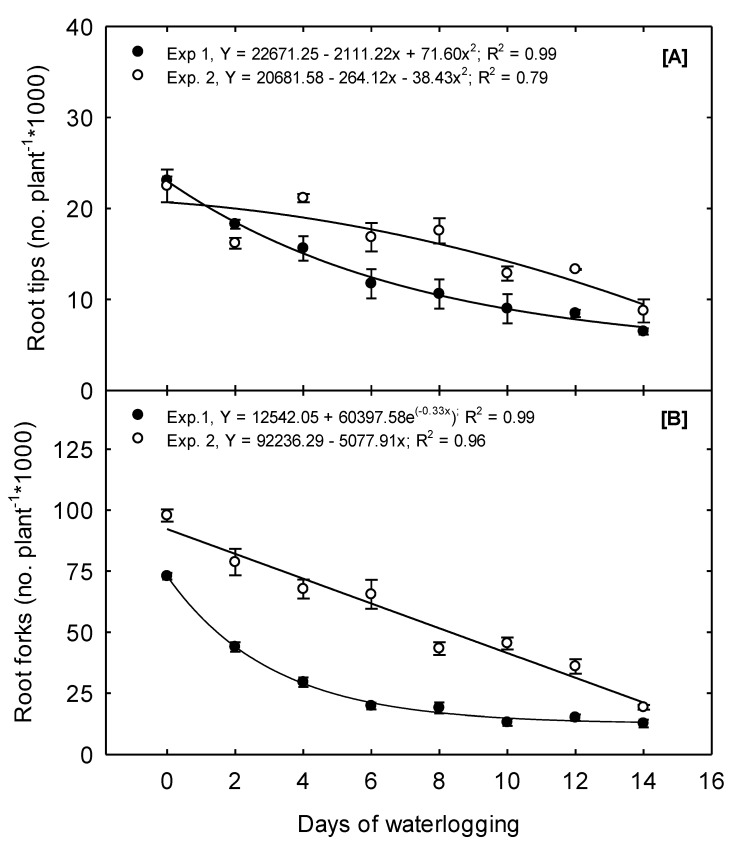
Waterlogging effects on root developmental parameters measured 15 days after waterlogging treatment. (**A**) Mean root tips show quadratic and convex quadratic decay in Exp. 1 and Exp. 2, respectively. (**B**) Mean root forks showing an exponential and linear decay in Exp.1 and Exp. 2, respectively. The values are the mean of six replications for each treatment.

In Exp. 1, 23,073 RT plant^−1^ were produced under the 0 days treatment. In contrast, 18,280, 10,604, and 6479 RT plant^−1^ were produced because of the 2, 8, and 14 days treatment, a decline of 20.8, 54, and 71.9% compared to the plant’s potential under the 0 days treatment—a concave quadratic trend best exemplified this declining trend (Figure 4A). In Exp. 2, RT plant^−1^ declined convex quadratically from a mean of 22,490 under the 0 days treatment to a mean of 8748 under the 14 days treatment. The impact of waterlogging on corn root tip numbers is not well documented. Still, declining trends like our observations of root tips have been reported in other crops such as wheat due to waterlogging [32].

Root forks are a good indicator of root branching and the complexity of the plant’s root system architecture. Root branching provides a means to increase the expanse of the soil that is reached and explored; additionally, branching can exponentially increase the number of root tips and the active absorptive surface area compared to root elongation alone [31]. In Exp. 1, an exponential decay function best described the relationship between waterlogging duration and RF (Figure 4B). Just two days of waterlogging resulted in a 40% reduction in root forks, but the number of RF under the 10, 12, and 14 days treatments did not differ (Table 2). Our observations align with previous reports of waterlogging significantly affecting a plant’s root system architecture [29].

Although plants are immediately hindered as root conductivity and metabolism decline under waterlogging conditions, reduced root growth and development can impact the plant through the entire growing season by restricting the water and nutrient uptake capacity [29]. In addition, Waterlogging often results in nitrogen (N) loss through excessive soil runoff and denitrification as soils become reduced under low oxygen environments [10]. Thus, corn plants with a weak root system face exacerbated issues obtaining adequate nitrogen as plants begin recovering post waterlogging and available nutrients are depleted. Such deficiencies could ultimately lead to further yield loss.

### 3.3. Leaf Pigments

A plant’s chlorophyll and polyphenol content are good indicators of a plant’s leaf N status; thus, these parameters can be used to interpret a plant’s ability to uptake nutrients through its root system. Chlorophyll (CHL) is the primary pigment responsible for leaf greenness, and close relationships have been established between leaf greenness and nitrogen (N) status. However, CHL estimation is not a perfect measure of N status, as leaf greenness can be affected by deficiencies of other nutrients such as sulfur, magnesium, and iron [33].

**Figure 5:**
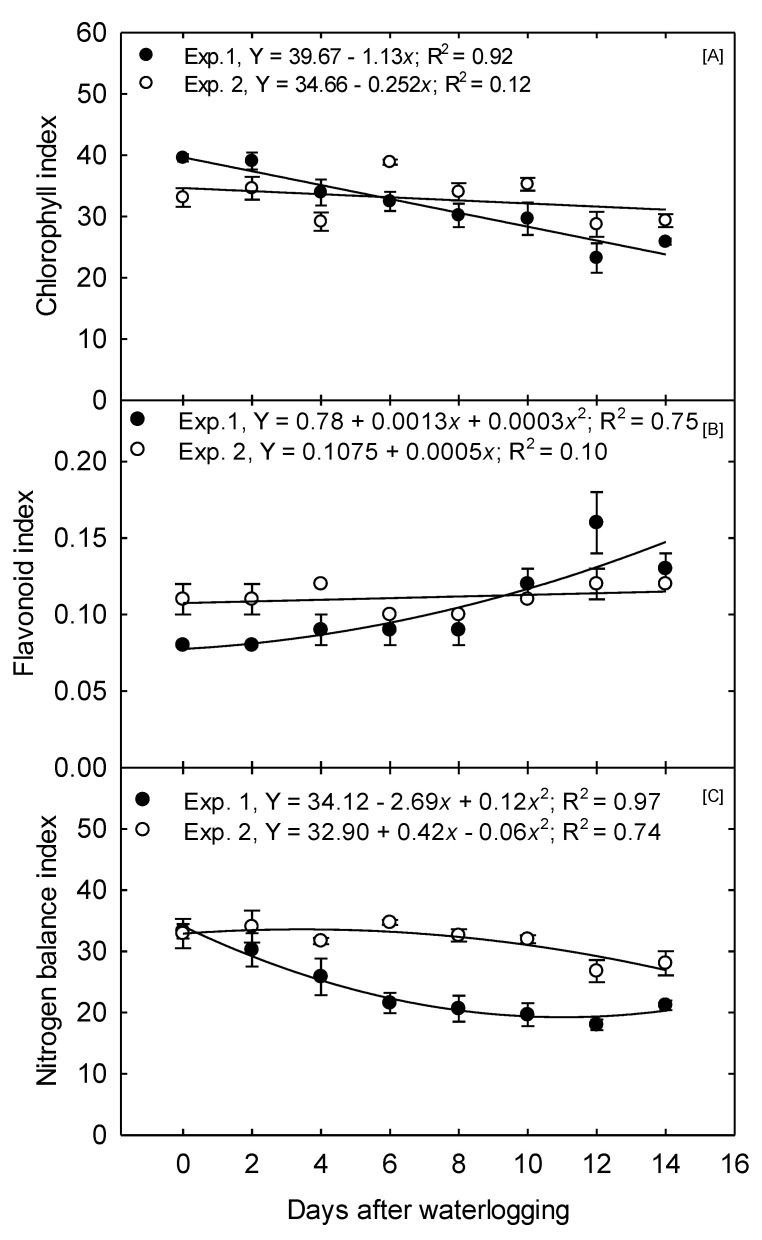
Waterlogging effect on corn physiological characteristics measured 15 days after waterlogging treatment. (**A**) Mean leaf chlorophyll (µg cm^−2^) showed a linear relationship for both experiments. (**B**) Mean anthocyanin values (unitless) index showing a quadratic and linear relationship for Exp. 1 and Exp. 2, respectively. (**C**) Mean nitrogen balance index (unitless) showing a quadratic and convex quadratic decay for Exp. 1 and Exp. 2, respectively. The values are the mean of six replications for each treatment.

In Exp. 1, CHL declined linearly as the duration of waterlogging treatments was prolonged, decreasing from 39.57, observed under the 0 days treatment, to 25.87, under the 14 days treatment; at a rate of 2.85% day^−1^, waterlogging treatments were extended (Figure 5A). In Exp. 2, no significant relationship was found between the increasing duration of waterlogging and CHL; however, CHL was lower under the 12- and 14 days treatments.

Anthocyanins (ANTH) are a good indicator that a plant is under stress. They serve as antioxidants to clean up free radicals which are generated because of plant stress. In a review from [34], anthocyanin production is suggested to be induced by environmental factors, including water stress. This production may be a protective mechanism for the plant to resist the harmful effects of such stress. In Exp. 1, ANTH did not differ within treatments ranging from 0 to 6 days (Table 2). However, a quadratic function best described the overall relationship between ANTH and the increasing duration of waterlogging, with the most significant increases occurring at higher treatment levels (Figure 5B). In Exp. 2, ANTH was only elevated due to the 12 and 14 days treatments. To our knowledge, no relationships between anthocyanin levels in corn and waterlogging have been established. However, previous research reports increased the levels of anthocyanins in some trees under waterlogged and flooded conditions resulting from the drought-like stress occurring due to a depressed root function [34].

The nitrogen balance index (NBI) estimates N status as a ratio of chlorophyll to polyphenolics. Research has shown that this ratio improves the indices’ relationship to crop N status compared to chlorophyll estimation alone [33]. For Exp 1, the NBI declined as treatments were prolonged from 0 to 6 days but ceased to decline significantly for treatments greater than 6 days (Table 2). Overall, the relationship between waterlogging duration and NBI best fits a quadratic function (Figure 5C). In Exp 2, NBI was only significantly lower under the 12 and 14 days treatments. In both studies, plants were treated with Hoagland’s nutrient solution, which always provided a readily available N source in the soil solution. Thus, declining leaf N could indicate an overall depressed root conductance. For a deficiency to become observable, the N uptake must be reduced to a greater relative extent than plant growth and demand.

### 3.4. Shoot Morphology

Vigorous shoot growth and leaf development during the early vegetative stages of corn are crucial to establishing a crop’s photosynthetic capacity and competitiveness against pests and weeds. These are both critical foundations of yield potential and stress tolerance throughout the growing season.

**Figure 6:**
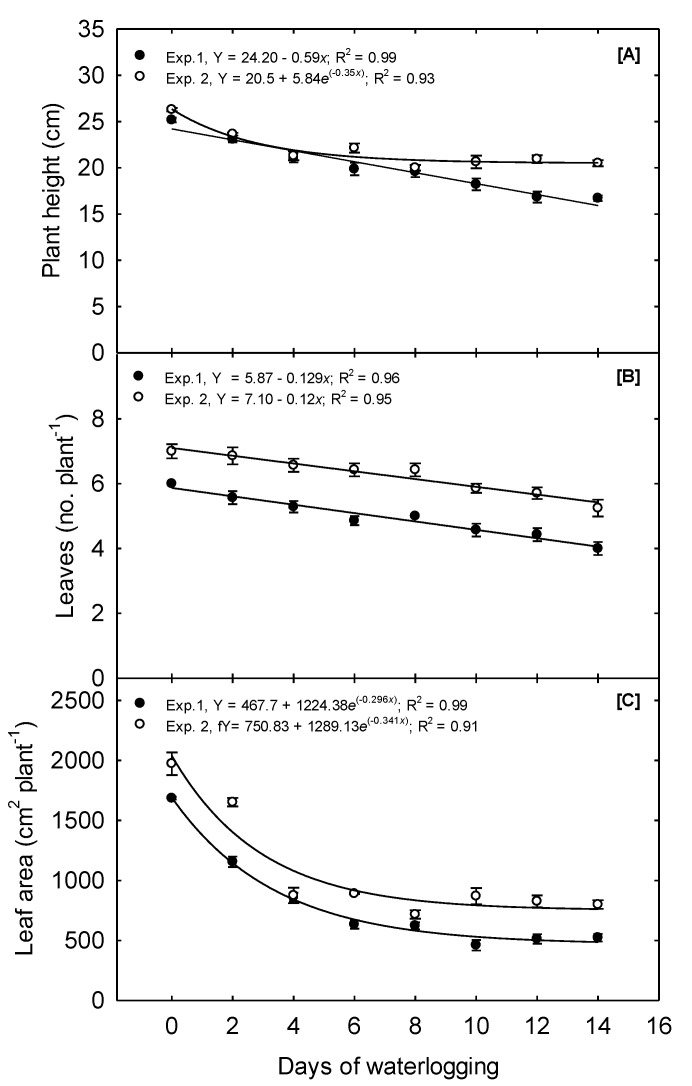
Waterlogging effect on corn shoot morphological parameters measured 15 days after waterlogging treatment. (**A**) Mean plant height showing linear and exponential decay for Exp. 1 and Exp. 2, respectively. (**B**) Mean leaf number showing linear decline for both experiments. (**C**) Mean whole-plant leaf area showing the exponential decay for both experiments. The values are the mean of six replications for each treatment.

In Exp. 1, plant height (PH) was reduced 4% day^−1^ of waterlogging as the duration increased from 0 to 4 days. Overall, plant height declined linearly from 25.17, observed at the 0 days treatment, to 16.71, observed under the 14d treatment, a total decline of 33.5% (Figure 6A). In Exp. 2, plant height declined 4.75% day^−1^ of waterlogging as the duration increased from 0 to 4 days; however, plant height under the 8, 10, 12, and 14 days treatments did not differ (Table 2). An exponential decay functions best described the relationship between waterlogging and PH. The mean plant height in this experiment was 26.29 when 0 days of waterlogging occurred and 20.5 when 14 days of waterlogging happened. Bragina et al. [35] reported that plant height declined 7, 11, and 23% when corn was flooded for 1, 2, and 3 days, respectively, at the V2 stage. Other studies have also reported a reduced plant height due to waterlogging in the field [3,6].

In Exp. 1, the leaf area per plant (LA) declined quadratically due to waterlogging increasing from 0 to 6 days; LA continued to decrease as treatments were prolonged past 6 days but at a much lower magnitude. A 31.5% decline in leaf area was observed due to just 2 days of waterlogging. The final leaf area was 1685.96 and 522.76 plant^−1^ for the 0 and 14 days treatments, respectively, a decline of 68.9%. Overall, exponential decay functions best describe the relationship between waterlogging and LA. In Exp. 2, the 2 days treatment reduced the leaf area by 16.3%, and the 4 days treatment reduced it by 55.5%. As the treatments were prolonged beyond 4 days, the final leaf area did not significantly decline further. The final leaf area was 1973.65 and 799.21 plant^−1^ for the 0 and 14 days treatment, respectively, constituting a total decline of 59.5%. Similar to Exp. 1, an exponential decay function best described the relationship between waterlogging and LA.

The number of leaves per plant (LN) was significantly affected by the increasing duration of waterlogging. The LN in Exp. 1 declined linearly from a mean of 6 leaves plant^−1^ to 4 leaves per plant as waterlogging treatments increased from 0 to 14 days, respectively. Exp. 2 displayed a similar linear trend, with LN declining from 7 to 5 under the 0 and 14 days treatment, respectively (Figure 6B). In this study, LN was measured as the number of leaves with a developed leaf collar. This measurement differs from the leaf initiation rate, a characteristic primarily controlled by temperature. Thus, waterlogging decreased the number of leaves with a developed collar, but we cannot determine how waterlogging affects the leaf initiation rate. To our knowledge, no previous reports outline the impacts of waterlogging on the leaf development or initiation rate.

### 3.5. Biomass Accumulation and Partitioning

A plant’s dry weight accumulation offers insight into its ability to convert sunlight into biomass. Additionally, we can determine how a plant prioritizes partitioning resources to different plant parts by measuring the dry weight accumulation of leaves, stems, and roots individually. The effects of increasing the duration of waterlogging on dry weight production and allocation followed a similar pattern during both experiments. Although leaf, stem, and root dry weight were all individually reduced by the treatments in both experiments, the root to shoot ratio and ratio of dry weight allocation to each part did not change due to the treatments. As waterlogging treatments increased in duration, the decline in the total plant dry weight was proportional to the decline observed for each individual component. However, contrary to our results, other studies have reported a decrease in the root to shoot ratio under 6d of waterlogging [18].

**Figure 7:**
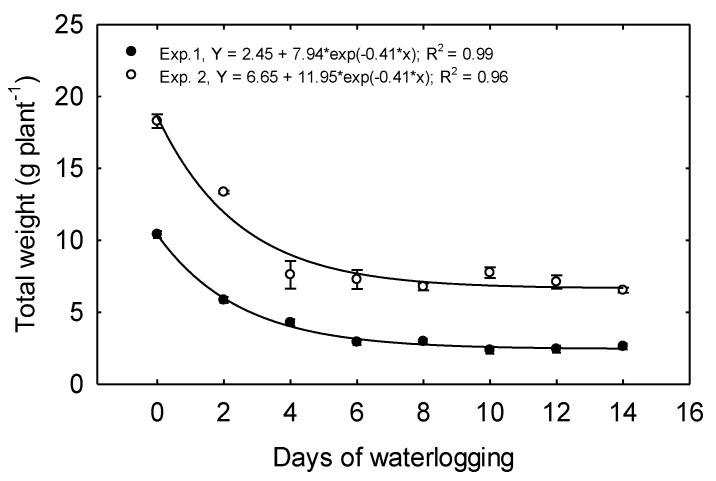
Waterlogging effect on dry weight accumulation measured 15 days after waterlogging treatment. Mean total dry weight showing the exponential decay for both experiments. The values are the mean of six replications for each treatment.

In Exp. 1, the total dry weight per plant (TDW) declined from 10.41 g plant^−1^ produced for 0 days treatment to 2.93 g plant^−1^ under 6 days treatment, a decline of 72% (Figure 7), and TDW was reduced by 43.8% due to just two days of waterlogging. Increasing the duration of waterlogging beyond 6 days did not incite further declines in biomass (Table 2).

In Exp. 2, TDW plant^−1^ declined as the treatment duration was prolonged from 0 to 4 days (Figure 7), and TDW plant^−1^ was reduced 27% due to just two days of waterlogging. No differences were observed in TDW under the 4 to14 days treatments.

In both experiments, exponential decay functions best described the relationship between TDW and waterlogging duration. Other studies have also shown a decrease in plant biomass due to waterlogging stress. When corn was grown in pots and flooded for 14 and 21 days, the biomass accumulation was reduced by more than 50% [6]. Lizaso and Ritchie [36] also reported a decreased biomass when corn was grown in pots and waterlogged for 6 days. Lizaso et al. [37] reported reduced biomass due to 4 and 8 days of waterlogging due to a reduced leaf area expansion and photosynthesis. In their experiment, doubling the duration of waterlogging from 4 to 8 days only decreased biomass by an additional 10%, suggesting a leveling-off response like that observed in this study. In contrast to our results, Wang et al. [38] found no differences in shoot biomass when corn seedlings were waterlogged for six days at the V2 growth stage.

Previous studies indicate that decreases in dry weight accumulation are likely due to multiple reactions from plants under stress, including reduced carbohydrate production and increased carbohydrate consumption. First, as root hydraulic conductivity declines, an increase in stomatal resistance quickly follows, limiting the uptake of water and nutrients. An internal water deficit can arise, which negatively affects photosynthesis at the leaf level. Further declines in photosynthesis can ensue as the leaf area is reduced, leaf senescence increases, and leaf chlorophyll declines. Second, as hypoxia is induced, plants transition their metabolism from aerobic respiration to fermentative metabolic routes. These are less efficient means of energy production. Glycolysis can increase as a compensatory reaction to fill this energy void, further depleting the carbohydrate reserves.

### 3.6. Water Stress Response Index (WSRI)

Waterlogging stress response indices were created for each measured parameter to illustrate the impact of waterlogging duration as a fraction of a plant’s potential under the 0 days treatment by normalizing the data as the relative response of each parameter to each treatment level as a fraction of the plant’s potential under the 0 days treatment.

**Figure 8:**
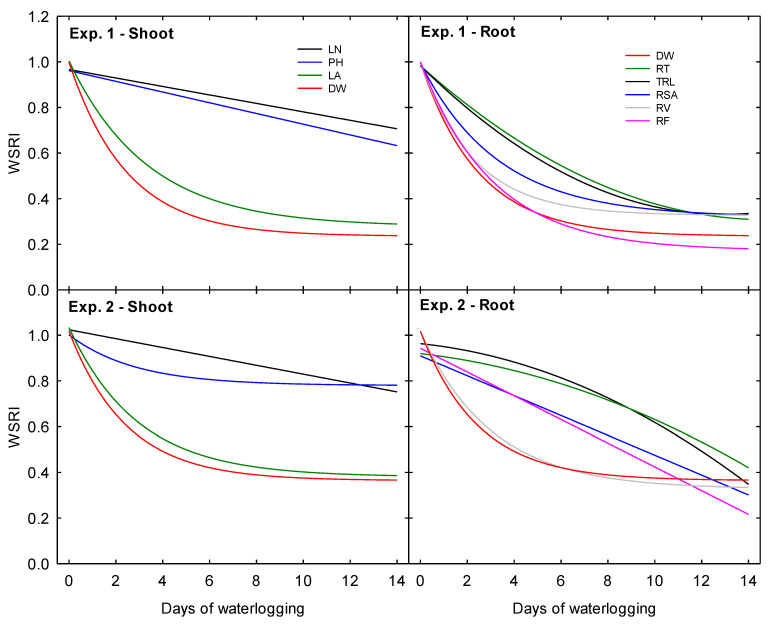
Waterlogging stress response indices for all shoot and root morphological parameters (LN—number of leaves, PH—plant height, LA—Leaf area, DW—plant dry weight components, RT—root tips, TRL—total root length, RAS—root surface area, RV—root volume, and RF—root forks). The values are derived by indexing each treatment’s mean to the mean of the 0 days (control) treatment. The curves represent fitted lines using linear, quadratic, and 3-parameter exponential decay functions. The constants for the regression functions are provided in Table 3.

In Exp. 1, LN and PH displayed the smallest relative decline. They were the only two morphological parameters in this experiment to hold a negative linear relationship with the increasing duration of waterlogging (Figure 8). Above the soil surface, TDW accumulation was the most inhibited parameter. An exponential decay functions best described the relationship between DW accumulation and treatment duration at the end of the experimental period. The sharpest relative declines occurred due to increasing the waterlogging time from 0 to 6 days. The relative decrease of LA, although not as severe, was closely correlated (R^2^ = 0.99) to that of DW. Below the soil surface, RV also declined in correlation with DW (R^2^ = 0.99). Like the relative response of LA, the decline was ultimately not as severe as it was for DW. Moreover, RSA declined in close correlation with DW (R^2^ = 0.99) and RV (R^2^ = 0.97); however, the relative rate of decline of RSA was less severe than that of RV. Ultimately, these two morphological traits declined to similar relative fractions under the 14 days treatment. The relative decrease in RF was also identical to that of DW. However, RF displayed the most significant relative decline under the 14 days treatment among all measured parameters. RT and TRL were closely correlated and showed the least relative reduction among all root parameters. Both RT and TRL declined following similar quadratic trends as the duration of waterlogging increased, with the most significant decline occurring as waterlogging increased from 0 to 6 days.

In Exp. 2, LN displayed the least relative decline as waterlogging treatments increased in duration (Figure 8). The response of PH followed closely behind, and the relative decline of these two parameters converged at the 12 days treatment. Above the soil surface, DW and LA were closely correlated (R^2^ = 0.99) and declined following similar exponential decay trends. Below the soil surface, DW and RV were also closely correlated (R^2^ = 0.99) and exhibited similar exponential decay trends. The relative response of RT and TRL were closely related (R^2^ = 0.99) and followed similar quadratic trends, with relative decline worsening as waterlogging treatments increased in duration. Finally, RSA and RF both declined following similar linear trends. Under the 14 days treatment, RF displayed the most significant relative decline among all parameters in this second experiment.

## 4. Conclusions

This study examined the impact of increasing durations of waterlogging from 0 to 14 days on corn’s early season growth and development. As waterlogging treatments were prolonged, the soil oxygen fraction began to rapidly decline until ultimately reaching zero around 10 and 8 days in Experiments 1 and 2, respectively. In both experiments, corn growth and development parameters were significantly impaired by waterlogging when measured 15 days after the treatments were imposed. Just two days of waterlogging reduced all parameters in comparison to the 0 days of waterlogging control. Linear, quadratic, and exponential decay functions were fit to describe the relationships between waterlogging duration and corn growth and development. Overall, plant height and leaf number were the least affected parameters under waterlogging stress. The dry weight biomass accumulation declined following an exponential decay function in both experiments as waterlogging duration was prolonged. After 14 days of waterlogging, root fork development appeared to be the most sensitive parameter to waterlogging. The relationships exhibited in this study will help exemplify the detrimental effects of waterlogging and for application in plant process and crop growth models and simulators.

## Figures and Tables

**Table 1:** Average air and soil temperatures and average daily solar radiation measured during the experimental period along with the standard errors of the mean.

Experiment	Air Temperature, °C	Soil Temperature, °C	Solar Radiation, MJ m^−2^ d^−1^
Exp. 1	24.73 ± 0.41	25.32 ± 0.14	17.72 ± 0.94
Exp. 2	27.85 ± 0.30	29.47 ± 0.25	26.68 ± 0.84

**Table 2:** Summary of the analysis of variance across waterlogging (W) treatments during each separate experiment on different growth, developmental, and physiological traits measured 15 days after the initial waterlogging treatment. Plant height (PH), number of leaves (LN), leaf area (LA), leaf dry weight (LDW), stem dry weight (StDW), root dry weight (RDW), whole-plant dry weight (TDW), root to shoot ratio (RS), total root length (TRL), root surface area (RSA), root volume (RV), root tips (RT), root fork (RF), chlorophyll content (CHL), flavonoids index (FI), anthocyanins index (ANTHI), and nitrogen balance index (NBI). Different lower-case letters within the columns denote a statistically significant difference between treatments according to the Fisher’s LSD test, ***, **, and NS indicates significance at *p* < 0.001, *p* < 0.05, and *p* < 0.05, respectively.

	Source	PH	LN	LA	LDW	StDW	RDW	TDW	RS	TRL	RSA	RV	RT	RF	CHL	FI	ANTHI	NBI
Experiment 1	Waterlogging (W)	***	***	***	***	***	***	***	NS	***	***	***	***	***	***	NS	***	***
	0	25.17a	6a	1685.96a	5.5a	3.63a	1.29a	10.41a	0.14	6166.07a	969.22a	12.15a	23,073.86a	72,940.29a	39.57a	1.2	0.08c	33.27a
	2	23.07b	5.57ab	1155.44b	3.23b	1.92b	0.7b	5.85b	0.14	4653.96b	648.9b	7.28b	18,280.71b	43,980.71b	39.06a	1.19	0.08c	30.25ab
	4	21.09c	5.29bc	861.76c	2.26c	1.52c	0.5c	4.28c	0.13	4233.95b	528.6c	5.53c	15,616.86b	29,493.29c	33.92b	1.42	0.09c	25.86bc
	6	19.86cd	4.86cde	633.24d	1.61d	0.97d	0.35d	2.93d	0.13	2979.22c	401.76d	4.32d	11,737.14c	19,746.29d	32.47b	1.28	0.09c	21.57dc
	8	19.57de	5cd	622.75ed	1.6d	0.98d	0.39cd	2.97d	0.15	2772.19cd	398.51d	4.57cd	10,604.14cd	18,963.14d	30.18cb	1.4	0.09c	20.64dc
	10	18.21ef	4.57de	460.89f	1.23e	0.85d	0.29d	2.37d	0.14	2062.44e	308.41e	3.7d	8981cde	13,079.29e	29.64cb	1.41	0.12b	19.65d
	12	16.83fg	4.43e	512.86f	1.3ed	0.8d	0.34d	2.44d	0.16	2320.99de	345.17de	4.11d	8467.14de	15,047de	23.22d	1.48	0.16a	18d
	14	16.71g	4e	522.96ef	1.38ed	0.92d	0.33d	2.63d	0.14	1949.52e	311.15e	4.02d	6479e	12,686.43e	25.87cd	1.35	0.13ab	21.2cd
Experiment 2	Waterlogging (W)	***	***	***	***	***	***	***	NS	***	***	***	***	***	***	NS	**	**
	0	26.29a	7a	1973.65a	9.29a	6.09a	2.92a	18.29a	0.19	6880.03a	1592.63a	29.9a	22,490.57a	97,822.57a	33.11bc	1.03	0.11bc	32.9a
	2	23.64b	6.86a	1652.53b	7.38b	3.76b	2.21b	13.35b	0.2	5762.15b	1281.06b	23.21b	16,187.29bc	78,707.43b	34.61b	1.04	0.11ab	34.03a
	4	21.29cd	6.57a	875.05c	3.8c	2.61c	1.2cde	7.61c	0.19	6652.06a	1005.45cd	10.48d	21,151.71a	67667.71c	29.16cd	0.93	0.12a	31.7ab
	6	22.14c	6.43ba	890.43c	2.86d	3.12cb	1.31c	7.28c	0.22	5453.88bc	1037.44c	14.72c	16,847.29b	65,497.29c	38.89a	1.12	0.1c	34.72a
	8	20ef	6.43ba	716.55d	2.84d	3.06cb	0.88e	6.77c	0.15	4840.98cd	782.54ef	10.13d	17,549b	43,266.29d	34.02b	1.04	0.1bc	32.61ab
	10	20.64de	5.86cb	869.18cd	3.82c	2.64c	1.29cd	7.75c	0.2	4224.39de	881.33de	14.75c	12,854d	45,405.71d	35.27ab	1.15	0.11bc	31.98ab
	12	20.93de	5.71c	826.59cd	3.33cd	2.79c	0.98de	7.1c	0.16	3712.01e	686.16f	10.26d	13,311.43cd	35,949.14d	28.73d	1.07	0.12a	26.79c
	14	20.5de	5d	799.21cd	3.29cd	2.28c	0.96e	6.53c	0.18	2209.62f	446.25g	9.23d	8748.43e	19,211e	29.34cd	1.06	0.12a	27.06c

**Table 3:** Regression parameters and coefficients of waterlogging stress response indices for all shoot and root morphological parameters of corn as affected by waterlogging duration [Y = y + ax for linear; Y = y0 + a ∗ x + b ∗ x^2^ for quadratic, and Y = y0 + a ∗ exp(−b ∗ x) for a 3-parameter exponential decay function, where Y is the plant parameter (LN—number of leaves, PH—plant height, LA—leaf area, TDW—whole plant dry weight, RT—root tips, RF—root fork, TRL—total root length, RV—root surface area, RV—root volume, and RF—root forks) and x the duration of waterlogging in days].

	y0	a	b	R^2^
**Exp. 1**				
LN ^1^	0.9664	−0.0186	--	0.9218
PH ^1^	0.9616	−0.0235	--	0.9539
LA ^3^	0.2774	0.7262	0.2963	0.9918
TDW ^3^	0.2354	0.7625	0.405	0.9953
RT ^3^	0.9826	−0.0915	0.0031	0.9893
TRL ^3^	0.9829	−0.1008	0.0039	0.9782
RSA ^3^	0.3189	0.6759	0.3003	0.9901
RV ^3^	0.3266	0.6724	0.4407	0.9936
RF ^3^	0.1719	0.828	0.3253	0.9964
**Exp. 2**				
LN ^1^	1.0239	−0.0194	--	0.897
PH ^3^	0.7796	0.2223	0.3549	0.929
LA ^3^	0.3804	0.6532	0.341	0.9121
TDW ^3^	0.3641	0.6532	0.4073	0.9583
TRL ^2^	0.9632	−0.0107	−0.0024	0.9411
RT ^2^	0.9196	−0.0117	−0.0017	0.7928
RSA ^1^	0.9095	−0.0435	--	0.9063
RF ^1^	0.9429	−0.0519	--	0.959
RV ^3^	0.3267	0.6894	0.3289	0.8511

^1^ Linear equation. ^2^ Quadratic equation. ^3^ Exponential decay function.

## Data Availability

Data is available from the author.

## Abbreviations

LMRAV	Lower Mississippi River Alluvial Valley
WSRI	Water stress response index
